# The prevalence of vertebral fractures in diffuse idiopathic skeletal hyperostosis and ankylosing spondylitis: A systematic review and meta-analysis

**DOI:** 10.1016/j.xnsj.2024.100312

**Published:** 2024-01-20

**Authors:** Netanja I. Harlianto, Solaiman Ezzafzafi, Wouter Foppen, Jonneke S. Kuperus, Irene E. van der Horst-Bruinsma, Pim A. de Jong, Jorrit-Jan Verlaan

**Affiliations:** a Department of Orthopedic Surgery, University Medical Center Utrecht and Utrecht University, Utrecht, the Netherlands; b Department of Radiology, University Medical Center Utrecht and Utrecht University, Utrecht, the Netherlands; c Department of Rheumatology, Radboud UMC, Nijmegen, The Netherlands

**Keywords:** Diffuse idiopathic skeletal hyperostosis, Ankylosing spondylitis, Spondylarthitis, Fracture, Vertebral fracture, Radiography, Computed tomography, Morbus bechterew, Morbus forestier

## Abstract

**Background:**

Subjects with ankylosing spinal disorders, including diffuse idiopathic skeletal hyperostosis (DISH) and ankylosing spondylitis (AS) are more prone to vertebral fractures and frequently present with neurological deficit compared to the patients without an ankylosed spine. Moreover, prevalent vertebral fractures are an important predictor for subsequent fracture risk. However, the pooled fracture prevalence for DISH is unknown and less recent for AS. We aimed to systematically investigate the prevalence and risk of vertebral fractures in DISH and AS populations.

**Methods:**

Publications in Medline and EMBASE were searched from January 1980 until July 2023 for cohort studies reporting vertebral fractures in AS and DISH. Data on prevalence were pooled with random effects modeling after double arcsine transformation. Heterogeneity was assessed with I^2^ statistics and we performed subgroup analysis and meta-regression to explore sources of heterogeneity.

**Results:**

We included 7 studies on DISH (n = 1,193, total fractures = 231) with a pooled vertebral fracture prevalence of 22.6% (95%CI: 13.4%–33.4%). For AS, 26 studies were included (n = 2,875, total fractures = 460) with a pooled vertebral fracture prevalence of 15.2% (95%CI: 11.6%–19.1%). In general, fracture prevalence for AS remained similar for several study-level and clinically relevant characteristics, including study design, diagnostic criteria, spine level, and patient characteristics in subgroup analysis. AS publications from 2010 to 2020 showed higher fracture prevalence compared to 1990 to 2010 (18.6% vs. 11.6%). Fractures in DISH were most common at the thoracolumbar junction, whereas for AS, the most common location was the mid-thoracic spine.

**Conclusions:**

Vertebral fractures are prevalent in AS and DISH populations. Differences in fracture distribution along the spinal axis exist between the 2 disorders. Additional longitudinal studies are needed for incident fracture assessment in patients with ankylosing spinal disorders.

## Introduction

Diffuse idiopathic skeletal hyperostosis (DISH) and ankylosing spondylitis (AS) are spinal disorders resulting in an increasingly rigid spine following progressive autofusion of vertebral bodies. AS and DISH patients are more at risk for vertebral fractures, even after minor trauma, as fused spinal segments are less flexible and incapable of appropriately distributing traumatic energy, with high local stress peaks as a consequence [1,2].

DISH is characterized by the formation of anterolateral bony bridges between vertebral bodies, and is most frequently observed in males and older populations [3]. While the exact mechanism of DISH remains inconclusive, DISH is frequently observed together with obesity, diabetes, and the metabolic syndrome, which suggests the involvement of metabolic and inflammatory processes in its pathogenesis [4,5]. Most frequent symptoms in DISH are back pain, but structures near the axial skeleton can also be compressed due to new bone formation, sometimes resulting in neurological deficit and symptoms including dyspnea and dysphagia [6].

In AS, chronic inflammation results in bone erosions and intravertebral bone loss, which is followed by new bone formation and subsequent fusion of vertebral bodies and facet joints. Men are most commonly affected, and the bony fusion of the spinal column often results in chronic inflammatory back pain. Moreover, extra-articular manifestations are frequently observed including peripheral entheses, uveitis, sacroiliitis, and generalized stiffness [7]. AS is most commonly classified using the (modified) New York criteria [8].

In 2017, a meta-analysis was published on risk factors of vertebral fracture in AS patients, queried until October 2015 [9]. The authors, however, did not assess the pooled prevalence of fractures and they included AS patients who did not always fulfill the (modified) New York criteria. Another meta-analysis focused on fractures in axial spondylarthritis between 2006 and 2016 [10]. Since then, several studies have been added to the literature [11,12]. Furthermore, a meta-analysis on vertebral fracture prevalence and risk in DISH has not yet been performed.

As patients with an ankylosed spine are a group at risk for spinal fractures, knowledge on the epidemiological prevalence can better inform clinicians and patients. Moreover, prevalent vertebral fractures are an important predictor for subsequent fracture risk, for which knowledge of the prevalence is important [13].

Hence, in the present meta-analysis, we aimed to assess the prevalence and risk factors of vertebral fractures in DISH, and aimed to reevaluate the state of evidence of prevalence and risk of vertebral fracture in AS, with additional analyses not conducted in prior meta-analyses.

## Methods

### Data sources and search strategy

This systematic review and meta-analysis was conducted according to the Preferred Reporting Items for Systematic Reviews and Meta-Analyses and Meta-Analysis of Observational Studies in Epidemiology guidelines (Appendix A & Appendix B) [14]. A systematic literature search was conducted in Medline and EMBASE from January 1, 1980 up until July 31, 2023 using a combination of the terms (“Ankylosing Spondylitis” AND “fracture”) OR (“DISH” AND “fracture”) with relevant synonyms. A detailed description of the full search is described in Appendix C. Language restrictions were not applied and we used cross-referencing to identify studies not included in the electronic search. Authors were not contacted for additional data.

### Study selection, data extraction and quality assessment

Title and abstract screening was independently performed by 2 investigators (N.I.H. & S.E.) for studies reporting vertebral fractures in AS and/or DISH populations. Disagreements and discrepancies between authors were discussed and resolved by consensus. For AS, we considered studies with unselected consecutive patients who were diagnosed according to the (modified) New York criteria, and we included DISH studies with patients classified according to Resnick criteria [8,15]. DISH is diagnosed following the presence of osseous bridging of at least 4 contiguous vertebrae; (relative) preservation of the intervertebral disc height; and the absence of apophyseal (facet) joint ankylosis or sacroiliac joint erosion. We excluded patient samples less than 10, as these were more likely to be case series than studies with consecutive patients.

One author (N.I.H) performed data extraction, which was checked with the original article by another reviewer (S.E.). Disagreements and discrepancies between authors were discussed and resolved by achieving consensus. Data were extracted on study design, year of publication, location, study period, mean age, percentage of males, body mass index (BMI), percentage diabetes, disease duration, imaging modality used, fracture assessment method, and spinal levels. For study quality assessment, we utilized the Joanna Briggs Institute critical appraisal tool for prevalence studies [16], which was independently performed by 2 authors, with agreement by consensus. This tool assesses the items regarding sampling frame, strategy, and size, the description of studies and setting, appropriate data analysis performance, reliable, and valid diagnosis, and response rate adequacy. We gave all studies an overall score, in accordance with the number of questions with a “Yes” response, for which the maximum score was equal to 9.

### Statistical analysis

The primary outcome of our study was the prevalence of vertebral fracture with 95% confidence intervals (95%CI) in DISH and AS populations. The pooled prevalence was obtained using the Freeman-Tukey double arcsine transformation to stabilize the variance of proportions [17]. To minimize the rates of between-study heterogeneity, summary estimates were pooled using random effect models. The extent of statistical heterogeneity was evaluated using Higgin's and Thompson's I^2^ [18]. If possible, potential sources of heterogeneity were explored, including study-level and clinically relevant characteristics such as year of publication, sex, age, spinal regions, and diagnostic criteria with stratified analyses and random effects meta-regression. In order to pool data of longitudinal studies in the prevalence meta-analysis, we included vertebral fracture data obtained at baseline, and not the cases who developed fractures during follow-up.

Publication bias was assessed using Egger's regression symmetry test [19]. Duval and Tweedie's nonparametric trim and fill method was used if there was evidence of publication bias [20]. The Mantel-Haenszel method was used to calculate the univariate prevalence odds ratio. Data analysis was performed with R version 4.1.3. (Foundation for Statistical Computing, Vienna, Austria) with the “meta” and “metafor” packages for meta-analysis.

## Results

### Study identification and characteristics

**Fig. 1:**
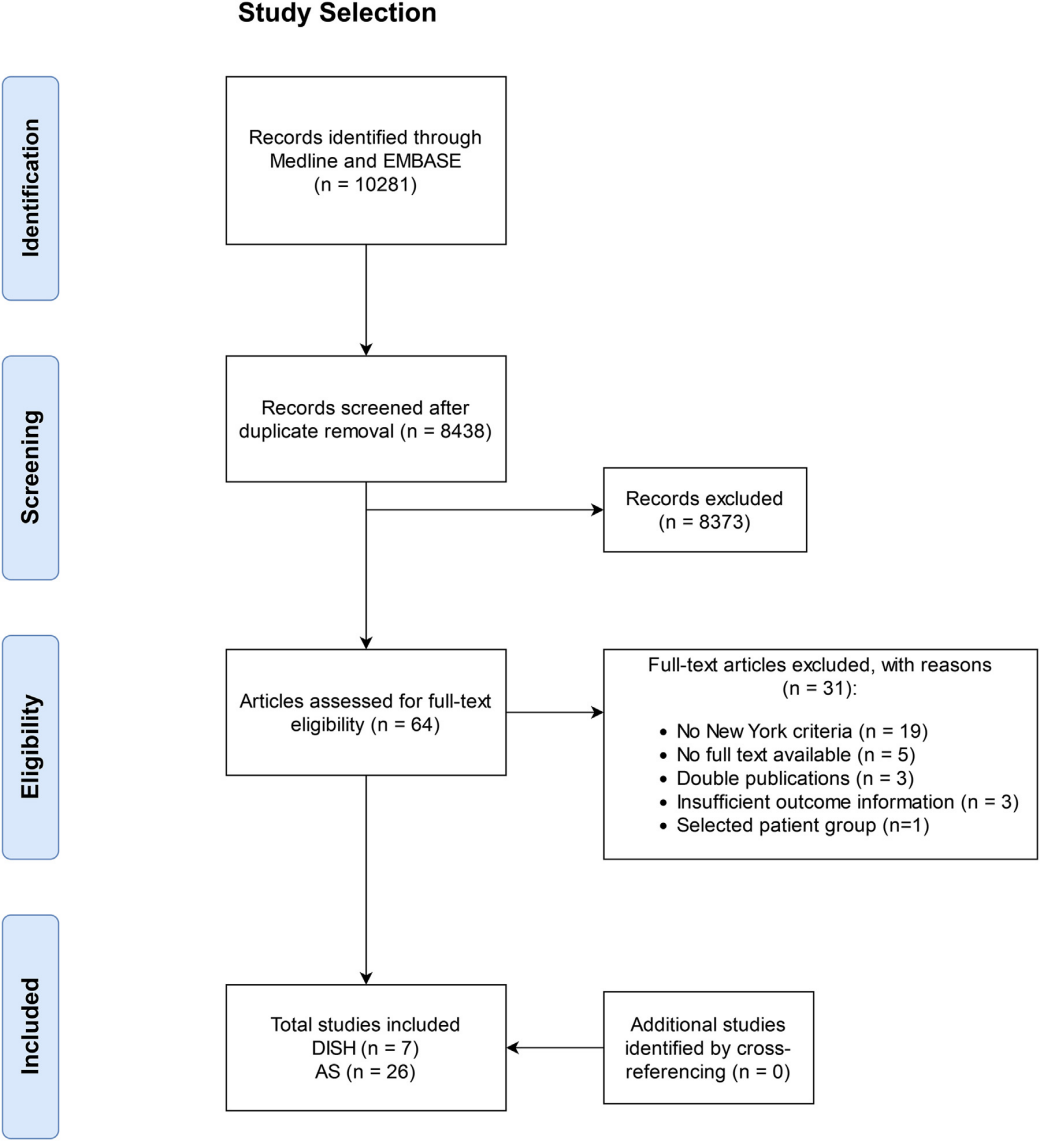
PRISMA flowchart of study selection.

A total of 8,438 articles were identified after duplicate removal. After title and abstract screening, 64 articles were assessed for full text eligibility, of which 31 were excluded with reason (Fig. 1). Finally, we included 7 DISH studies and 26 AS studies in the meta-analysis [11], [12], [21], [22], [23], [24], [25], [26], [27], [28], [29], [30], [31], [32], [33], [34], [35], [36], [37], [38], [39], [40], [41], [42], [43], [44], [45], [46], [47], [48], [49], [50], [51]. Study characteristics of DISH studies are shown in Table 1. For DISH, the assessment of vertebral fractures was cross-sectional in 6 studies and longitudinal in 1 study. Five studies used radiographs and 2 studies used CT for fracture evaluation, and the Genant method for fracture assessment was reported by 6 studies. Fractures at the thoracolumbar spine were reported by 6 studies, whereas 1 study focused solely on the thoracic spine. Mean ages for DISH patients ranged from 67 to 78.6 years, diabetes ranged from 14% to 24.5%, and 5 out of 6 studies reported a mean BMI of 28 kg/m^2^ or higher.

**Table 1:** Study characteristics DISH.

Author, y, JBI	Country	Design	Period	Spine level	Imaging	Fracture assessment	Mean age (SD)	% Male	% Diabetes	Mean BMI (SD)	DISH cases	No. with VF	% VF
Pini, 2023 [21], 9	Spain	Cross-sectional	NA	T-L	Radiograph	Genant	66.8 (9)	0	17	30.9 (8)	126	36	28.6
Furukawa 2022, 7 [22]	Japan	Cross-sectional	2008–2018	T-L	CT	NS	78.6 (NS)	70	NS	NS	140	52	37.1
Guiot 2021, 9 [23]	France	Longitudinal	1995–2004	T-L	Radiograph	Genant	68 (7)	100	15.2	29.5 (3.9)	164	12	7.3
Pini 2021, 9 [24]	Spain	Cross-sectional	NS	T-L	Radiograph	Genant	67 (15)	100	22.2	29.9 (4.4)	207	43	20.8
Watanabe 2020, 6 [25]	Japan	Cross-sectional	2016–2019	T-L	CT	Genant	76.6 (5)	100	24.5	22.8 (3.1)	49	20	40.8
Katzman 2017, 8 [26]	USA	Cross-sectional	1986–2002	T	Radiograph	Genant	71.6 (NS)	58.7	12.7	29 (NS)	378	33	8.7
Diederichs 2011, 9 [27]	USA	Cross-sectional	2000–2002	T-L	Radiograph	Genant	75.2 (6.2)	100	15	28.1 (3.5)	129	35	27.1

JBI, Johanna Briggs Institute quality score; VF, vertebral fracture; SD, standard deviation; NS, not specified; T, thoracic; L, lumbar;

Genant = Genant et al. [72].

For AS, 4 studies assessed vertebral fractures longitudinally (15.4%), whereas the majority of studies were cross-sectional in design (84.6%) (Table 2). Radiographs were used in all studies, most frequently according to the Genant method (19/25, 73.1%) for fracture assessment. Most of the studies evaluated the thoracolumbar spine (73.1%), whereas 4 studies assessed fractures in the cervical, thoracic, and lumbar spine (15.4%), and 3 studies in the lumbar spine only (11.5%). Described AS patient populations were predominantly male (median: 79.9%; IQR: 70.6%–87.7%), with a median average age of 39.9 years (IQR: 36.8–43.9 years) and a median average disease duration of 11.5 years (IQR: 9.8–16.0 years). The included studies were generally considered to have good quality, as the mean average critical appraisal score across all studies was 7.8 out of 9 (Appendix D).

**Table 2:** Study characteristics AS.

Author, y, JBI	Country	Design	Period	Spine level	Imaging	Fracture assessment	AS criteria	Mean age (SD)	% Male	Disease duration years (SD)	AS cases	No. with VF	% VF
Kim 2022, 7 [11]	Korea	Cross-sectional	2012–2020	T-L	Radiograph	NS	MNY	47.6 (13.8)	66	4.1 (3.9)	219	20	9.1
Fauny 2021, 7 [12]	France	Cross-sectional	2009–2017	C-T-L	Radiograph	Genant	MNY	60.3 (10.7)	89	24 (12–34)*	73	9	12.3
Beek 2019, 9 [28]	Netherlands	Longitudinal	2003–2014	T-L	Radiograph	Genant	MNY	42.8 (10.2)	70	11.9 (9.5)	135	15	11.1
Maas 2017, 9 [29]	Netherlands	Longitudinal	2004–2012	T-L	Radiograph	Genant	MNY	42.8 (12.5)	70	16 (8-25)*	292	59	20.0
Van der Weijden 2016, 8 [30]	Netherlands	Longitudinal	NS	T-L	Radiograph	Genant	MNY	41.8 (9.2)	82	12.2 (9.1)	49	6	12.2
Rossini 2016, 8 [31]	Italy	Cross-sectional	2012–2014	T-L	Radiograph	Genant	MNY	47 (NS)	83	11.5 (NS)	71	18	25.4
Kang 2014, 9 [32]	Korea	Longitudinal	2007–2013	L	Radiograph	Genant	MNY	33.9 (10.9)	80	3.8 (5.1)	298	31	10.8
Ulu 2013, 8 [34]	Turkey	Cross-sectional	2007–2011	T-L	Radiograph	Genant	MNY	34.5 (9)	80	4.8 (4.8)	86	24	27.9
Ulu 2013, 8 [35]	Turkey	Cross-sectional	2011–2012	T-L	Radiograph	Genant	MNY	34.3 (9.4)	85	11.5 (7.5)	59	18	30.5
Klingberg 2012, 8 [36]	Sweden	Cross-sectional	2009–2009	C-T-L	Radiograph	Genant	MNY	50 (13)	57	24 (13)	204	24	11.8
Montala 2012, 8 [33]	Spain	Cross-sectional	NS	T-L	Radiograph	Genant	MNY	48.6 (13.1)	78	22.5 (12.6)	176	57	32.4
Arends 2011, 9 [37]	Netherlands	Cross-sectional	2004–2009	T-L	Radiograph	Genant	MNY	41 (11.1)	73	14 (NS)	128	41	32
Mermerci 2010, 8 [38]	Turkey	Cross-sectional	NS	T-L	Radiograph	>15% any height	MNY	39.9 (10.9)	75	10.5 (7.8)	100	19	19
Ghozlani 2009, 9 [39]	Morocco	Cross-sectional	2007–2008	T-L	Radiograph	Genant	MNY	38.9 (11.8)	84	10.8 (6.6)	80	34	42.5
Caglayan 2007, 7 [40]	Turkey	Cross-sectional	NS	L	Radiograph	Genant	MNY	36.8 (NS)	100	7.6 (6.8)	38	8	21.1
Jun 2006, 8 [41]	Korea	Cross-sectional	2004–2004	T-L	Radiograph	Genant	MNY	30.7 (6.5)	100	7.2 (5.4)	68	11	16.2
Lange 2005, 6 [42]	Germany	Cross-sectional	NS	T-L	Radiograph	Genant	NY	44 (NS)	63	19.5 (NS)	84	9	10.7
Baek 2004, 8 [43]	Korea	Cross-sectional	1997–1998	C-T-L	Radiograph	Genant	MNY	28.1 (7.9)	100	9.4 (5.1)	76	3	3.9
Maillefert 2001, 8 [44]	France	Cross-sectional	NS	T-L	Radiograph	Genant	MNY	37.3 (11.3)	65	12.4 (8.6)	54	2	3.7
Toussirot 2001, 7 [45]	France	Cross-sectional	1997–1999	L	Radiograph	Genant	MNY	39.1 (11.5)	69	10.6 (8.3)	71	1	1.4
Mitra 2000, 7 [46]	England	Cross-sectional	NS	T-L	Radiograph	McCloskey	MNY	37.8 (NS)*	100	9.9 (NS)*	66	11	16.7
Sivri 1996, 6 [47]	Turkey	Cross-sectional	NS	T-L	Radiograph	>20% for T and >15% for L	NY	36.8 (6.3)	91	9.8 (6.8)	22	9	40.9
Donnelly 1994, 7 [48]	England	Cross-sectional	NS	T-L	Radiograph	McCloskey	NY	43.9 (NS)	71	16.4 (NS)	87	8	9.2
Cooper 1994, 8 [49]	USA	Cross-sectional	1935–1989	T-L	Radiograph	Radiologist report	MNY	33.8 (12.3)	77	NS	158	15	9.5
Devogelaer 1992, 6 [50]	Belgium	Cross-sectional	NS	T-L	Radiograph	Genant	NY	38.4 (NS)	86	15.1 (NS)	70	3	4.3
Ralston 1990, 8 [51]	Scotland	Cross-sectional	NS	C-T-L	Radiograph	>20% for T and >15% for L	MNY	41 (NS)	88	17 (NS)	111	20	18

JBI, Johanna Briggs Institute quality score; VF, vertebral fracture; SD, standard deviation; NS, not specified; C, cervical; T, thoracic; L, lumbar; MNY, modified New-York criteria; NY, New-York criteria.

⁎ median (interquartile range). Genant = Genant et al. [72]; McCloskey = McCloskey et al. [73]

### Prevalence of vertebral fractures in DISH

**Fig. 2:**
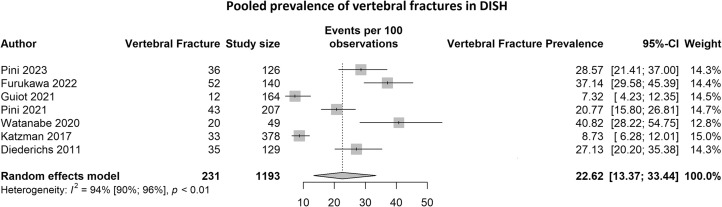
The pooled vertebral fracture prevalence for DISH.

**Fig. 3:**
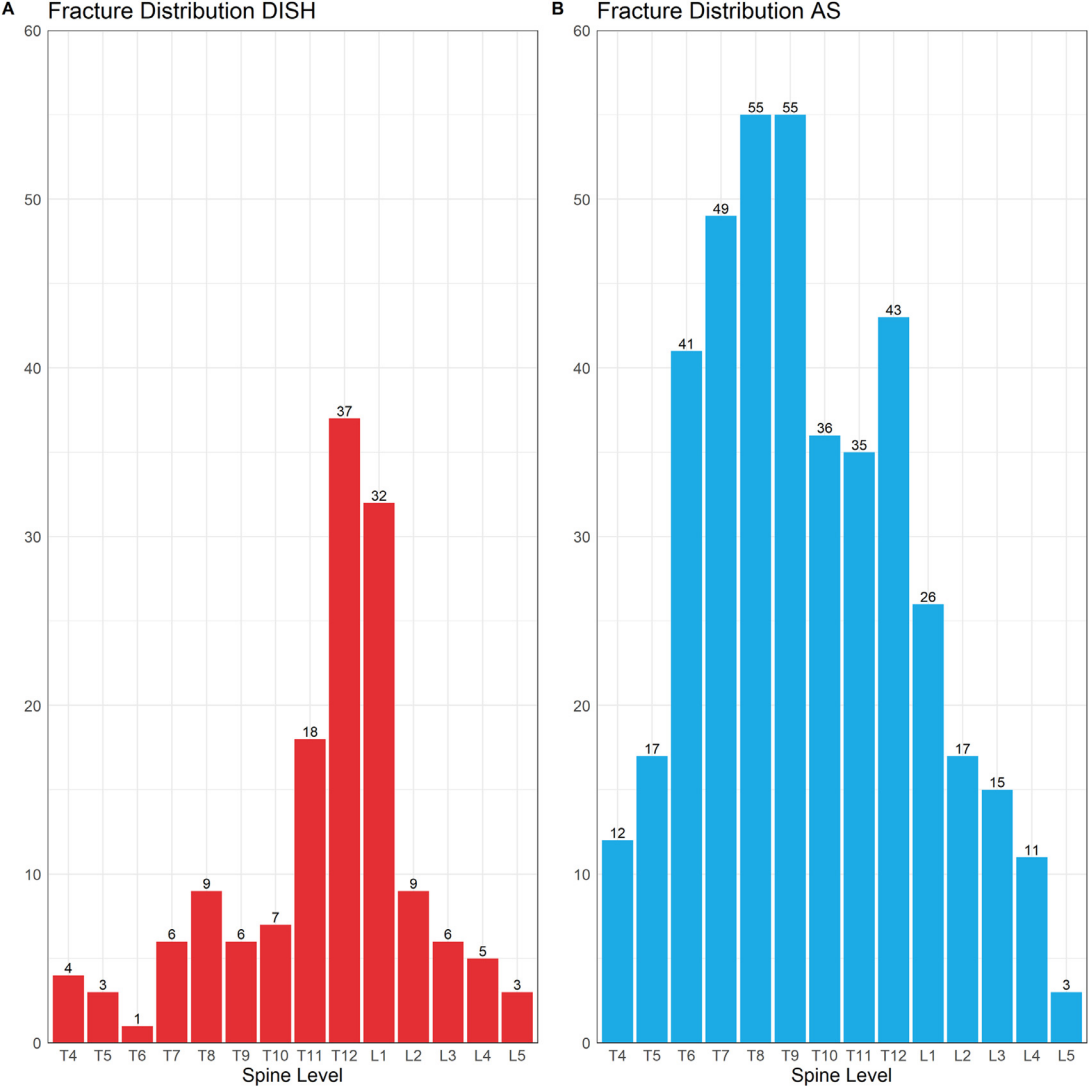
(A) The distribution of compression fracture by spine levels in AS. (B) The distribution of compression fractures by spine level in DISH.

Seven studies reporting vertebral fracture prevalence in DISH were identified comprising 1193 DISH patients, of which 231 patients had vertebral fractures. The pooled vertebral fracture prevalence was 22.6% (95%CI: 13.4%–33.4%) and there was evidence of significant heterogeneity I^2^ = 94% (95%CI: 90%–96%, p < 0.01) (Fig. 2). As the number of studies was less than 10, publication bias was not assessed. The pooled vertebral fracture prevalence remained similar when only including studies that used the Genant method (20.4%; 95%CI: 11.1%–31.6%. I^2^ = 93%; 95%CI: 87%–96%,p < 0.01). Three studies [20,22,26] described the fracture distribution by spine level (Fig. 3A); a total of 146 vertebral fractures in 97 patients were reported, with most fractures observed in the thoracolumbar junction (T12-L1) (47.3%).

### Fracture risk in DISH

One study reported the risk for vertebral fracture risk in DISH patients compared to controls based on prevalence data. A cross-sectional study in prostate cancer patients reported an adjusted OR of 5.99 (95%CI: 2.16–16.6) after adjustments for BMI and BMD [25]. We calculated the univariate odds ratios for fracture risk between DISH and controls for 5 other cross-sectional studies with available data using the Mantel-Haenszel method. No significant differences in vertebral fracture risk were found between DISH and controls in the cohorts of Pini et al. [21], [24]], (OR 1.89; 95%CI: 1.27-2.82 and OR 1.05; 95%CI: 0.72-1.52), Katzman et al. [26] (OR 0.73; 95%CI: 0.50-1.07), and Diederichs et al. [27] OR 1.20; 95%CI: 0.74-1.96). Data on fracture risk were not pooled due to the heterogeneity of patient sample sizes, different patient and location demographics, and the limited number of studies (Appendix E).

### Prevalence of vertebral fractures in AS

**Fig. 4:**
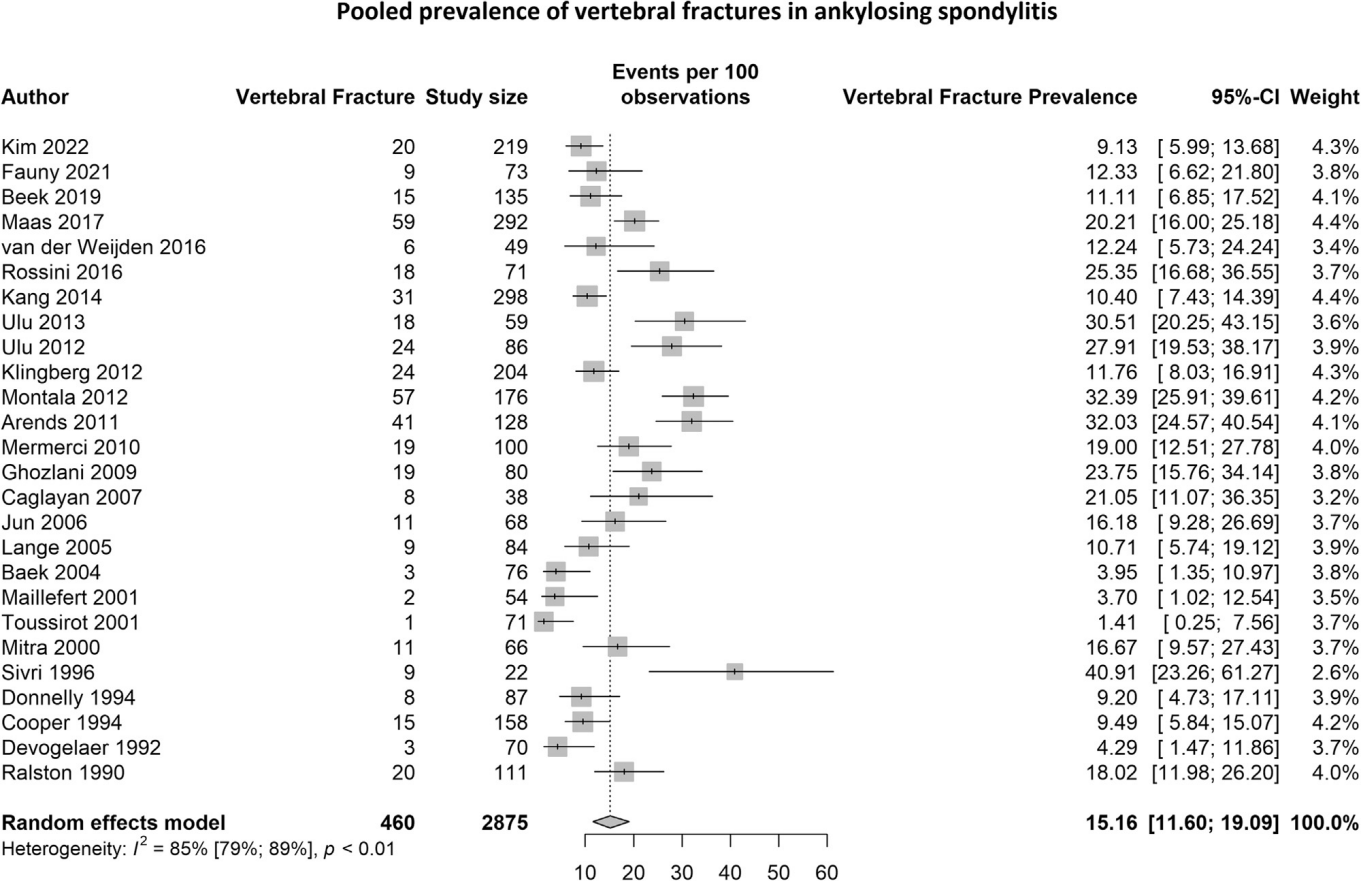
The pooled vertebral fracture prevalence for AS.

We identified 26 studies encompassing a total of 2875 AS patients, of which 460 patients had vertebral fractures. The pooled vertebral fracture prevalence was 15.2% (95%CI: 11.6% - 19.1%) and statistical heterogeneity was 85% (95%CI: 79% - 89%, p<0.01) (Figure 4). Egger's regression test was not significant (p=0.76), indicating that there was no evidence of publication bias. Pooled fracture prevalence remained similar after excluding 3 studies who used the New York criteria. Subgroup analyses and meta-regression analyses are shown in Appendix F. Heterogeneity was not explained by study design, diagnostic criteria, spine level, and patient characteristics (*p-value* for meta-regression > 0.10). AS publications from 2010-2022 showed higher fracture prevalence compared to 1990-2010 (18.6% vs. 11.6%, meta-regression p=0.055).

### Fracture distribution and severity in AS

The distribution of fractures was reported in 7 studies (415 fractures in 254 patients) [[28], [29], [30],[33], [36], [39], [51]. Most vertebral fractures were observed in the mid-portion of the thoracic spine T6-T9 (48.2%) (Figure 3B). The severity of vertebral fractures using the Genant method was reported in 8 studies [12,28,29,33,36,37,39,44] comprising 423 vertebral fractures in 260 patients. The sum of fractures stratified by severity was mild (20-25% reduction) for 291 fractures (68.8%), moderate (25-40% reduction) for 118 fractures (27.9%), and severe (>40% reduction) for 14 fractures (3.3%).

## Discussion

### Main findings

In this meta-analysis, we aimed to determine the pooled vertebral fracture prevalence in DISH and AS populations. In line with previous work [10], our results indicate that subjects with AS have a vertebral fracture prevalence of approximately 15%, with the addition of several relevant findings which have not been addressed previously. The estimated fracture prevalence is less than 5% for non-ankylosed spines under 60 years [52]. We explored the vertebral fracture prevalence in patients with AS without restricting the year of publication. Our results displayed higher vertebral fracture prevalence in the last decade in comparison to papers published before 2010, though no other patient or study-level characteristics explaining heterogeneity were identified. This may be attributable to increased diagnostic accuracy of radiography from film to digital technology, the natural progressive disease course of AS, and/or the increased awareness of vertebral fracture risk in AS populations.

For DISH, we found that subjects with DISH have an approximate vertebral fracture prevalence of 21.4%, which has not been systematically reported previously. It is important to note that the fracture prevalence for AS and DISH were derived from asymptomatic populations, and not from the traumatic setting. It is estimated that vertebral fracture prevalence between 60-80 years of age are between 5.4-10.5% in patients without an ankylosed spine [52]. To date, there have been a limited number of published studies assessing prevalent vertebral fractures and fracture risk in DISH and 1 study concluded that DISH is associated with an increased risk for incident vertebral fractures [23]. With regards to prevalent vertebral fractures, the available evidence is still rather limited with a wide confidence interval for DISH and with heterogeneous patient populations. It is apparent that many publications have been published focusing on vertebral fracture prevalence in AS the last decades from 1990 onwards, whereas publications on vertebral fracture prevalence in DISH are more recent and limited in number, and most likely more sensitive imaging modalities.

### Similarities and differences between DISH and AS

Fracture severity was only reported for AS, where 70% of vertebral fractures were classified as mild (compression: 20-25%) and 28% as moderate (compression: 25-40%). For fracture distribution we were able to compare AS and DISH and we observed differences between the populations in our study. In DISH, compression fractures were predominantly located at the thoracolumbar junction, which was less pronounced in AS, as most fractures were observed along the thoracic spine in AS. The fusion of vertebral bodies results in a stiff spine [53], which increases vertebral fracture risk. It is important to note that for AS, radioprogression scores, including the modified Stoke Ankylosing Spondylitis Spinal Score (mSaSSS) includes the lateral view of the cervical and lumbar spine, which may lead to underdiagnosis of fractures [54]. Patients with DISH were much older compared to patients with AS in our included studies. Advancing age is an important risk factor for vertebral fracture risk, which may influence vertebral fracture risk in DISH and AS [55]. Increased BMI has also been associated with an increased risk for vertebral fractures, though this was not always observed in male populations [56]. It has been well established that AS patients have an increased prevalence of osteoporosis [10], [57], whereas several studies in DISH patients have found comparable or higher BMD values in DISH compared to non-DISH patients [26], [58], [59]. Hence, fracture risk in DISH is predominantly most likely due to the increased energy stress peaks and less due to BMD levels, whereas osteoporosis plays an important role in fracture risk in AS.

Moreover, it is hypothesized that inflammation plays a role in the process of bone formation in DISH and AS [60], [61], and there is emerging evidence that inflammatory markers are associated with clinically relevant vertebral fractures [62]. Both AS and DISH patients have been linked with an increased cardiovascular risk profile, further supporting the role of inflammation in the diseases [63], [64], [65]. In addition, patients with DISH have more type 2 diabetes compared to patients without DISH, with the reported proportion in our study between 13 and 25%. The presence of diabetes has been associated with an increased risk of incident vertebral fractures [66].

Extensive research has been performed on fall risk in AS, with medical factors including poor balance, gait, and mobility, fear of falling, active disease, and symptoms contributing to this risk [67]. In contrast, risks of falls have not yet been explored in patients with DISH to our best knowledge, though it can be postulated that the high age and spinal stiffness may play a role herein as well. Differences also exist in non-vertebral fracture risk between AS and DISH. Non-vertebral fracture risk was increased in AS patients in a previous meta-analysis [9]. The longitudinal study by Guiot et al. [23] was the only study to evaluate this risk, and found no non-vertebral fracture risk in DISH patients after adjustments for age, BMI, femoral BMD, previous fracture, calcification, disc space narrowing, and endplate irregularities.

The increased prevalence of vertebral fractures in these populations, compared to patients without ankyloses, highlights the importance of awareness and vertebral fracture prevention.

Treatment for vertebral compression fractures encompasses conservative treatment in the form of analgesics such as NSAIDs and opioids, and fracture prevention with bisphosphonates and calcitonin. However, in the presence of mechanical instability, surgical treatment is preferred. Instrumentation for vertebral fracture fixation is usually performed, with the level of fusion extending 2 to 3 levels below and above the fracture [68], [69]. Latest AS guidelines do not mention bisphosphonates, denosumab, or TNF-alpha inhibitors treatment for vertebral fracture prevention [70]. For DISH, osteoporosis and/or anti-inflammatory treatment for fracture prevention remains an area to be further explored in future research.

### Strengths and limitations

Our review has several strengths. First, compared to previous AS literature, we included more than twice the number of studies and the number of patients in our meta-analysis with our comprehensive and unrestricted literature search. Second, we performed detailed exploratory analyses by patient characteristics and study level qualities to identify sources of heterogeneity and whether publication bias was present. Third, the inclusion of both DISH and AS allowed for the comparison and discussion of patient and disease characteristics between the 2 entities. However, our study also has limitations. We could not perform detailed subgroup analyses by relevant characteristics for DISH given the limited data and number of studies. Future studies reporting vertebral fractures in DISH and AS should adhere to reporting these based on AO Spine Classifications [71]. Moreover, recent nomenclature of axial spondylitis also encompasses a non-radiographic disease form, which we did not include in our analyses [70]. Finally, few studies reported fracture prevalence in “healthy” controls, which restricted the pooling of vertebral fracture risk in DISH patients compared with controls. Though, to our best knowledge, this is the first study to systematically investigate the literature on vertebral fracture prevalence in DISH. Our results are comprehensive and important given the high presentation with neurological deficits, and future pseudoarthrosis and vertebral fracture risk [13], though they should be construed in the context of the available level of evidence. Additional research assessing longitudinal fracture risk in DISH cohorts is warranted.

## Conclusion

Aggregated published data suggest that vertebral fractures are prevalent in patients with AS and DISH in unselected patients not in the trauma setting. For DISH, around the average age of 70 years, approximately 22.6% of patients had vertebral fractures. For AS, around the average age of 40 years, 15.2% of patients had vertebral fractures.

Differences exist between DISH and AS regarding fracture distribution along the spine, as fractures in DISH were most common at the thoracolumbar junction, which was observed in the mid-thoracic spine for AS.

## Data availability statement

The datasets generated during and/or analyzed during the current study are available from the corresponding author on reasonable request.

## Declaration of competing interest

The authors declare that they have no known competing financial interests or personal relationships that could have appeared to influence the work reported in this paper.

## Appendix Supplementary materials

Image, application 1
