# Supplementary material for: The prevalence of vertebral fractures in diffuse idiopathic skeletal hyperostosis and ankylosing spondylitis: A systematic review and meta-analysis
Source: N Am Spine Soc J. 2024 Jan 20;17:100312. doi: 10.1016/j.xnsj.2024.100312 (PMC10869944; doi:10.1016/j.xnsj.2024.100312)
Supplement: Supplementary file 1 [file mmc1.docx]

**The prevalence of vertebral fractures in diffuse idiopathic skeletal hyperostosis and ankylosing spondylitis: A systematic review and meta-analysis**

**Table of contents:**

1. Appendix A: PRISMA checklist
2. Appendix B: MOOSE checklist
3. Appendix C: Electronic Pubmed & Embase search
4. Appendix D: Quality assessment using the Joanna Briggs Institute critical appraisal tool for prevalence studies
5. Appendix E: Forest plot odds ratio DISH
6. Appendix F: Subgroup analyses and meta-regression analyses

**Appendix A. PRISMA checklist**

| **Section and Topic** | **Item #** | **Checklist item** | **Location where item is reported** |
| --- | --- | --- | --- |
| **TITLE** | | |  |
| Title | 1 | Identify the report as a systematic review. | Page 1 |
| **ABSTRACT** | | |  |
| Abstract | 2 | See the PRISMA 2020 for Abstracts checklist. | Page 1 |
| **INTRODUCTION** | | |  |
| Rationale | 3 | Describe the rationale for the review in the context of existing knowledge. | Pages 2-5 |
| Objectives | 4 | Provide an explicit statement of the objective(s) or question(s) the review addresses. | Pages 2-5 |
| **METHODS** | | |  |
| Eligibility criteria | 5 | Specify the inclusion and exclusion criteria for the review and how studies were grouped for the syntheses. | Page 6 |
| Information sources | 6 | Specify all databases, registers, websites, organisations, reference lists and other sources searched or consulted to identify studies. Specify the date when each source was last searched or consulted. | Page 6 |
| Search strategy | 7 | Present the full search strategies for all databases, registers and websites, including any filters and limits used. | Supplementary Table S1. |
| Selection process | 8 | Specify the methods used to decide whether a study met the inclusion criteria of the review, including how many reviewers screened each record and each report retrieved, whether they worked independently, and if applicable, details of automation tools used in the process. | Pages 6-7 |
| Data collection process | 9 | Specify the methods used to collect data from reports, including how many reviewers collected data from each report, whether they worked independently, any processes for obtaining or confirming data from study investigators, and if applicable, details of automation tools used in the process. | Pages 6-7 |
| Data items | 10a | List and define all outcomes for which data were sought. Specify whether all results that were compatible with each outcome domain in each study were sought (e.g. for all measures, time points, analyses), and if not, the methods used to decide which results to collect. | Pages 6-7 |
|  | 10b | List and define all other variables for which data were sought (e.g. participant and intervention characteristics, funding sources). Describe any assumptions made about any missing or unclear information. | Pages 6-7 |
| Study risk of bias assessment | 11 | Specify the methods used to assess risk of bias in the included studies, including details of the tool(s) used, how many reviewers assessed each study and whether they worked independently, and if applicable, details of automation tools used in the process. | Page 6-7, Supplementary Table S2 |
| Effect measures | 12 | Specify for each outcome the effect measure(s) (e.g. risk ratio, mean difference) used in the synthesis or presentation of results. | Pages 6-7 |
| Synthesis methods | 13a | Describe the processes used to decide which studies were eligible for each synthesis (e.g. tabulating the study intervention characteristics and comparing against the planned groups for each synthesis (item #5)). | Pages 6-7 |
|  | 13b | Describe any methods required to prepare the data for presentation or synthesis, such as handling of missing summary statistics, or data conversions. | Not applicable |
|  | 13c | Describe any methods used to tabulate or visually display results of individual studies and syntheses. | 6-7 |
|  | 13d | Describe any methods used to synthesize results and provide a rationale for the choice(s). If meta-analysis was performed, describe the model(s), method(s) to identify the presence and extent of statistical heterogeneity, and software package(s) used. | 7 |
|  | 13e | Describe any methods used to explore possible causes of heterogeneity among study results (e.g. subgroup analysis, meta-regression). | 7 |
|  | 13f | Describe any sensitivity analyses conducted to assess robustness of the synthesized results. | 7 |
| Reporting bias assessment | 14 | Describe any methods used to assess risk of bias due to missing results in a synthesis (arising from reporting biases). | Supplementary Table S2 |
| Certainty assessment | 15 | Describe any methods used to assess certainty (or confidence) in the body of evidence for an outcome. | Supplementary Table S2 |
| **RESULTS** | | |  |
| Study selection | 16a | Describe the results of the search and selection process, from the number of records identified in the search to the number of studies included in the review, ideally using a flow diagram. | Figure 1, page 8 |
|  | 16b | Cite studies that might appear to meet the inclusion criteria, but which were excluded, and explain why they were excluded. | Figure 1 |
| Study characteristics | 17 | Cite each included study and present its characteristics. | 8 |
| Risk of bias in studies | 18 | Present assessments of risk of bias for each included study. | Supplementary Table S2 |
| Results of individual studies | 19 | For all outcomes, present, for each study: (a) summary statistics for each group (where appropriate) and (b) an effect estimate and its precision (e.g. confidence/credible interval), ideally using structured tables or plots. | Page 9-10, Figure 2 and 4 |
| Results of syntheses | 20a | For each synthesis, briefly summarise the characteristics and risk of bias among contributing studies. | Supplementary Table S2 |
|  | 20b | Present results of all statistical syntheses conducted. If meta-analysis was done, present for each the summary estimate and its precision (e.g. confidence/credible interval) and measures of statistical heterogeneity. If comparing groups, describe the direction of the effect. | Page 9-10, Figure 2 and 4 |
|  | 20c | Present results of all investigations of possible causes of heterogeneity among study results. | Page 9-10, Figure 2 and 4 |
|  | 20d | Present results of all sensitivity analyses conducted to assess the robustness of the synthesized results. | Supplementary Figure S2-S9 |
| Reporting biases | 21 | Present assessments of risk of bias due to missing results (arising from reporting biases) for each synthesis assessed. | Supplementary Table S2 |
| Certainty of evidence | 22 | Present assessments of certainty (or confidence) in the body of evidence for each outcome assessed. | Supplementary Table S2 |
| **DISCUSSION** | | |  |
| Discussion | 23a | Provide a general interpretation of the results in the context of other evidence. | Page 11 |
|  | 23b | Discuss any limitations of the evidence included in the review. | Page 13 |
|  | 23c | Discuss any limitations of the review processes used. | Page 13 |
|  | 23d | Discuss implications of the results for practice, policy, and future research. | Page 12-14 |
| **OTHER INFORMATION** | | |  |
| Registration and protocol | 24a | Provide registration information for the review, including register name and registration number, or state that the review was not registered. | Not registered |
|  | 24b | Indicate where the review protocol can be accessed, or state that a protocol was not prepared. |  |
|  | 24c | Describe and explain any amendments to information provided at registration or in the protocol. |  |
| Support | 25 | Describe sources of financial or non-financial support for the review, and the role of the funders or sponsors in the review. | Not applicable |
| Competing interests | 26 | Declare any competing interests of review authors. | Uploaded COI forms |
| Availability of data, code and other materials | 27 | Report which of the following are publicly available and where they can be found: template data collection forms; data extracted from included studies; data used for all analyses; analytic code; any other materials used in the review. | Page 14 |

*From:*  Page MJ, McKenzie JE, Bossuyt PM, Boutron I, Hoffmann TC, Mulrow CD, et al. The PRISMA 2020 statement: an updated guideline for reporting systematic reviews. BMJ 2021;372:n71. doi: 10.1136/bmj.n71

For more information, visit: <http://www.prisma-statement.org/>

**Appendix B:**MOOSE Checklist:

The prevalence of vertebral fractures in diffuse idiopathic skeletal hyperostosis and ankylosing spondylitis: a systematic review and meta-analysis

| **Criteria** | | **Brief description of how the criteria were handled in the review** |
| --- | --- | --- |
| **Reporting of background** | |  |
| √ | Problem definition | Subjects with ankylosing disorders, including DISH and AS have an increased risk for vertebral fractures. This is important given the increased presentation with neurological deficit in traumatic settings, and the risk for future vertebral fractures. |
| √ | Hypothesis statement | Fracture rates differ for AS and DISH populations, including spinal and patient characteristics |
| √ | Description of study outcomes | Rates of vertebral fracture |
| √ | Type of exposure | The presence of an ankylosed spine, either diffuse idiopathic skeletal hyperostosis or ankylosing spondylitis |
| √ | Type of study designs used | Cross-sectional and longitudinal studies (retrospective, prospective, or randomized controlled trials) |
| √ | Study population | Generally unselected patients with radiologically confirmed vertebral fractures |
| **Reporting of search strategy should include** | |  |
| √ | Qualifications of searchers | Netanja I. Harlianto, MD-PhD Student; Solaiman Ezzafzafi, BSc, MD-Student |
| √ | Search strategy, including time period included in the synthesis and keywords | Time period: From January 1980 until December, 2022.  The detailed search strategy can be found in Supplementary Table S1. |
| √ | Databases and registries searched | MEDLINE, EMBASE |
| √ | Search software used, name and version, including special features | EndNote was used to manage references |
| √ | Use of hand searching | We cross-referenced bibliographies of included papers |
| √ | List of citations located and those excluded, including justifications | Details of the literature search process are outlined in the flow chart Figure 1. |
| √ | Method of addressing articles published in languages other than English | We applied no language restrictions |
| √ | Method of handling abstracts and unpublished studies | We did not contact investigators for unpublished data or unpublished abstracts. Abstracts were excluded (Figure 1.) |
| √ | Description of any contact with authors | We did not contact authors of studies |
| **Reporting of methods should include** | |  |
| √ | Description of relevance or appropriateness of studies assembled for assessing the hypothesis to be tested | Inclusion and exclusion criteria are specified in the Methods section. |
| √ | Rationale for the selection and coding of data | Data extracted from each of the studies were relevant to the population characteristics, study design, exposure, and outcome. |
| √ | Assessment of confounding | We included only studies where populations were unselected |
| √ | Assessment of study quality, including blinding of quality assessors; stratification or regression on possible predictors of study results | Study quality was assessed based on the Johanna Briggs Critical Appraisal Checklist for Studies Reporting Prevalence Data, a validated instrument which is designed for assessment of methodological quality of prevalence studies |
| √ | Assessment of heterogeneity | We quantified heterogeneity of the studies with I^2^ statistic that provides the relative amount of variance of the summary effect due to the between-study heterogeneity. Moreover, heterogeneity was explored using meta-regression and stratified analyses if possible |
| √ | Description of statistical methods in sufficient detail to be replicated | The methods section details the statistics used for meta-analyses, sensitivity analyses, meta-regression and assessment of publication bias. We performed random effects meta-analysis with R version 4.1.3. |
| √ | Provision of appropriate tables and graphics | Tables 1-2 and; Figs. 2,3,4; Supplementary Figures S1-S9 |
| **Reporting of results should include** | |  |
| √ | Graph summarizing individual study estimates and overall estimate | Figs. 2,4 |
| √ | Table giving descriptive information for each study included | Table 1 and Table 2 |
| √ | Results of sensitivity testing | We conducted sensitivity analysis to assess the influence of some large studies. This was done by omitting such studies and calculating a pooled estimate for the remainder of the studies |
| √ | Indication of statistical uncertainty of findings | 95% confidence intervals were presented with all summary estimates, I^2^ values and results of sensitivity analyses |
| **Reporting of discussion should include** | |  |
| √ | Quantitative assessment of bias | Sensitivity analyses indicate heterogeneity in strengths of the association due to most common biases in observational studies. The systematic review is limited in scope, as it involves published data. More high quality studies in DISH are needed. Limitations have been discussed. |
| √ | Justification for exclusion | All studies were excluded based on the pre-defined inclusion criteria in methods section. |
| √ | Assessment of quality of included studies | Brief discussion included in ‘Methods’ section |
| **Reporting of conclusions should include** | |  |
| √ | Consideration of alternative explanations for observed results | Discussed in the Discussion section |
| √ | Generalization of the conclusions | Discussed in the context of the results. |
| √ | Guidelines for future research | We recommend evidence from additional large longitudinal studies |
| √ | Disclosure of funding source | In “Source of Funding” section |

**Appendix C: Supplementary Table S1. Electronic database searches**

| **Database** |  |
| --- | --- |
| PUBMED | **#1 DISH** Hyperostosis, Diffuse Idiopathic Skeletal[MeSH] OR Diffuse Idiopathic Skeletal Hyperostosis[tiab] OR Vertebral Ankylosing Hyperostosis[tiab] OR Forestier’s Disease[tiab] OR Forestiers Disease[tiab] OR Forestier-Rotes-Querol [tiab] OR Ankylosing hyperostosis[tiab] OR spondylosis hyperostotica[tiab] OR Senile ankylosing spondylitis[tiab] OR Disseminated idiopathic skeletal hyperostosis[tiab] OR Cervical hyperostosis[tiab] OR osteophyte[tiab] OR ostheophytes[tiab] OR spondylophytes[tiab] OR anterior hyperostosis[tiab] OR anterior longitudinal ligament[tiab] OR senile ankylosing hyperostosis[tiab] OR senile ankylosing vertebral hyperostosis[tiab] OR coating vertebral hyperostosis[tiab] OR Rotes-Querol[tiab] OR ossification anterior longitudinal ligament[tiab] OR ossification ALL[tiab] OR OALL[tiab] OR calcification of the anterior longitudinal ligament[tiab] OR calcification anterior ligament[tiab] OR calcification longitudinal ligament[tiab] OR spinal hyperostosis[tiab] OR spinal osteophyte[tiab] OR spinal osteophytes[tiab] OR osteophytes of the spine[tiab]  **#2 AS**  Spondylitis, Ankylosing[MeSH] OR Spondyloarthritis[tiab] OR spondylarthritis[tiab] OR ankylosing spondylitis[tiab] OR ankylosing spondylarthritis[tiab] OR  ankylosing spondyloarthritis[tiab] OR  ankylosing spondylarthritides[tiab] OR  ankylosing spondyloarthritides[tiab] OR  spondyloarthritis ankylopoietica[tiab] OR  spondylarthritis ankylopoietica[tiab] OR  rheumatoid spondylitis[tiab] OR  spondylitis ankylosans[tiab] OR  spondylarthritis ankylopoetica[tiab] OR  spondyloarthritis ankylopoetica[tiab] OR  spondylitis deformans[tiab] OR  spondylitis adolescens[tiab] OR  spondylitis ankyloarthritica[tiab] OR spondylitis atrophica ligamentosa[tiab] OR  spondylitis ossificans ligamentosa[tiab] OR  Bechterew[tiab] OR  Bechterew disease[tiab] OR  Bechterews disease[tiab] OR  Bechterew syndrome[tiab] OR  Bechterew arthritis[tiab] OR  morbus Bechterew[tiab] OR  bamboo spine[tiab] OR Bekhterev[tiab] OR Bekhterev disease[tiab] OR  Marie-Struempell disease[tiab] OR Marie Struempell disease[tiab] OR  Marie disease[tiab] OR  Marie-Strümpell disease[tiab] OR Pierre Marie disease[tiab] OR HLA-B27[tiab]  **#3 Fracture**  trauma[tiab] OR spinal fractures[tiab] OR vertebral fracture[tiab] OR fractured[tiab] OR fractures[tiab] OR fractures, bone[MeSH] OR fracture[tiab] OR spine fracture[tiab] OR Osteoporosis [MeSH] OR osteoporotic fractures[MeSH] OR osteoporosis [tiab] OR fractures[tiab] OR osteoporo*[tiab] OR burst spinal fracture[tiab] OR burst fracture[tiab] OR Multiple Trauma[MeSH] OR polytrauma[tiab]  Search: (#1 AND #3) OR (#2 AND #3) |
| EMBASE | **#1 DISH** 'ankylosing hyperostosis'/exp  OR ‘Diffuse Idiopathic Skeletal Hyperostosis':ti,ab,kw OR ‘Vertebral Ankylosing Hyperostosis':ti,ab,kw OR ‘Forestiers Disease':ti,ab,kw  OR ‘Forestier-Rotes-Querol':ti,ab,kw  OR ‘Ankylosing hyperostosis':ti,ab,kw  OR ‘spondylosis hyperostotica':ti,ab,kw  OR ‘Senile ankylosing spondylitis':ti,ab,kw  OR ‘Disseminated idiopathic skeletal hyperostosis':ti,ab,kw  OR ‘Cervical hyperostosis':ti,ab,kw  OR ‘osteophyte':ti,ab,kw  OR ‘ostheophytes':ti,ab,kw  OR ‘spondylophytes':ti,ab,kw  OR ‘anterior hyperostosis':ti,ab,kw  OR ‘anterior longitudinal ligament':ti,ab,kw  OR ‘senile ankylosing hyperostosis':ti,ab,kw  OR ‘senile ankylosing vertebral hyperostosis':ti,ab,kw  OR ‘coating vertebral hyperostosis':ti,ab,kw OR ‘Rotes-Querol':ti,ab,kw OR ‘ossification anterior longitudinal ligament':ti,ab,kw OR ‘ossification ALL':ti,ab,kw  OR ‘OALL':ti,ab,kw OR ‘calcification of the anterior longitudinal ligament':ti,ab,kw OR ‘calcification anterior ligament':ti,ab,kw OR ‘calcification longitudinal ligament':ti,ab,kw OR ‘spinal hyperostosis':ti,ab,kw OR ‘spinal osteophyte':ti,ab,kw OR ‘spinal osteophytes':ti,ab,kw OR ‘osteophytes of the spine':ti,ab,kw  **#2 AS**  'ankylosing spondylitis'/exp OR ‘Spondyloarthritis':ti,ab,kw OR ‘spondylarthritis':ti,ab,kw OR ‘ankylosing spondylitis':ti,ab,kw OR ‘ankylosing spondylarthritis':ti,ab,kw OR ‘ankylosing spondyloarthritis':ti,ab,kw OR ‘ankylosing spondylarthritides':ti,ab,kw OR ‘ankylosing spondyloarthritides':ti,ab,kw OR ‘spondyloarthritis ankylopoietica':ti,ab,kw OR  ‘spondylarthritis ankylopoietica':ti,ab,kw OR ‘rheumatoid spondylitis':ti,ab,kw OR ‘spondylitis ankylosans':ti,ab,kw OR ‘spondylarthritis ankylopoetica':ti,ab,kw OR ‘spondyloarthritis ankylopoetica':ti,ab,kw OR ‘spondylitis deformans':ti,ab,kw OR ‘spondylitis adolescens':ti,ab,kw OR ‘spondylitis ankyloarthritica':ti,ab,kw OR ‘spondylitis atrophica ligamentosa':ti,ab,kw OR ‘spondylitis ossificans ligamentosa':ti,ab,kw OR ‘Bechterew':ti,ab,kw OR ‘Bechterew disease':ti,ab,kw OR ‘Bechterews disease':ti,ab,kw OR  ‘Bechterew syndrome':ti,ab,kw OR ‘Bechterew arthritis':ti,ab,kw OR ‘morbus Bechterew':ti,ab,kw OR ‘bamboo spine':ti,ab,kw OR ‘Bekhterev':ti,ab,kw OR ‘Bekhterev disease':ti,ab,kw OR ‘Marie-Struempell disease':ti,ab,kw OR ‘Marie Struempell disease':ti,ab,kw OR ‘Marie disease':ti,ab,kw OR ‘Marie-Strümpell disease':ti,ab,kw OR ‘Pierre Marie disease':ti,ab,kw OR ‘HLA-B27':ti,ab,kw  **#3 Fracture**  ‘trauma':ti,ab,kw OR ‘spinal fractures':ti,ab,kw OR ‘vertebral fracture':ti,ab,kw OR ‘fractured':ti,ab,kw OR ‘fractures':ti,ab,kw OR ‘fracture':ti,ab,kw OR ‘spine fracture':ti,ab,kw OR ‘osteoporosis':ti,ab,kw OR ‘fractures':ti,ab,kw OR ‘osteoporo*':ti,ab,kw OR ‘burst spinal fracture':ti,ab,kw OR ‘burst fracture':ti,ab,kw OR ‘polytrauma':ti,ab,kw OR 'injury'/exp OR ‘injury':ti,ab,kw OR 'fracture'/exp OR ‘fracture':ti,ab,kw OR 'spine fracture'/exp OR 'spine fracture':ti,ab,kw OR 'fragility fracture'/exp OR 'fragility OR fracture':ti,ab,kw OR 'multiple trauma'/exp OR 'multiple trauma':ti,ab,kw  Search: (#1 AND #3) OR (#2 AND #3) |

**Appendix D: Supplementary Table S2. Quality assessment of included studies**

| Study | Was the sample frame appropriate to address the target population? | Were study participants sampled in an appropriate way? | Was the sample size adequate? | Were the study subjects and the setting described in detail? | Was the data analysis conducted with sufficient coverage of the identified sample? | Were valid methods used for the identification of the condition? | Was the condition measured in a standard, reliable way for all participants? | Was there appropriate statistical analysis? | Was the response rate adequate, and if not, was the low response rate managed appropriately? | Overall |
| --- | --- | --- | --- | --- | --- | --- | --- | --- | --- | --- |
| Pini et al. (2023) | 1 | 1 | 1 | 1 | 1 | 1 | 1 | 1 | 1 | 9 |
| Furukawa et al. (2022) | 1 | 1 | 1 | 0 | 1 | 0 | 1 | 1 | 1 | 7 |
| Guiot et al. (2021) | 1 | 1 | 1 | 1 | 1 | 1 | 1 | 1 | 1 | 9 |
| Pini et al. (2021) | 1 | 1 | 1 | 1 | 1 | 1 | 1 | 1 | 1 | 9 |
| Watanabe (2020) | 1 | 0 | 0 | 1 | 1 | 1 | 1 | 1 | 0 | 6 |
| Katzman et al. (2017) | 1 | 1 | 1 | 1 | 1 | 1 | 1 | 1 | 0 | 8 |
| Diederichs et al. (2017) | 1 | 1 | 1 | 1 | 1 | 1 | 1 | 1 | 1 | 9 |
| Kim et al. (2022) | 1 | 0 | 1 | 1 | 1 | 0 | 1 | 1 | 1 | 7 |
| Fauny et al. (2021) | 1 | 1 | 0 | 0 | 1 | 1 | 1 | 1 | 1 | 7 |
| Beek et al. (2019) | 1 | 1 | 1 | 1 | 1 | 1 | 1 | 1 | 1 | 9 |
| Maas et al. (2017) | 1 | 1 | 1 | 1 | 1 | 1 | 1 | 1 | 1 | 9 |
| Van der Weijden et al. (2016) | 1 | 1 | 0 | 1 | 1 | 1 | 1 | 1 | 1 | 8 |
| Rossini et al. (2016) | 1 | 1 | 0 | 1 | 1 | 1 | 1 | 1 | 1 | 8 |
| Kang et al. (2014) | 1 | 1 | 1 | 1 | 1 | 1 | 1 | 1 | 1 | 9 |
| Ulu et al. (2013) | 1 | 1 | 1 | 1 | 1 | 1 | 1 | 0 | 1 | 8 |
| Ulu et al. (2013) | 1 | 1 | 1 | 1 | 1 | 1 | 1 | 0 | 1 | 8 |
| Klingberg et al. (2012) | 1 | 0 | 1 | 1 | 1 | 1 | 1 | 1 | 1 | 8 |
| Montala et al. (2012) | 1 | 1 | 1 | 1 | 1 | 1 | 1 | 1 | 0 | 8 |
| Arends et al. (2011) | 1 | 1 | 1 | 1 | 1 | 1 | 1 | 1 | 1 | 9 |
| Mermerci et al. (2010) | 1 | 0 | 1 | 1 | 1 | 1 | 1 | 1 | 1 | 8 |
| Ghozlani et al. (2009) | 1 | 1 | 1 | 1 | 1 | 1 | 1 | 1 | 1 | 9 |
| Caglayan et al. (2007) | 1 | 0 | 0 | 1 | 1 | 1 | 1 | 1 | 1 | 7 |
| Jun et al. (2006) | 1 | 1 | 0 | 1 | 1 | 1 | 1 | 1 | 1 | 8 |
| Lange et al. (2005) | 1 | 0 | 1 | 0 | 1 | 1 | 1 | 0 | 1 | 6 |
| Baek et al. (2004) | 1 | 1 | 1 | 1 | 1 | 1 | 1 | 0 | 1 | 8 |
| Maillefert et al.(2001) | 1 | 1 | 0 | 1 | 1 | 1 | 1 | 1 | 1 | 8 |
| Toussirot et al. (2001) | 1 | 1 | 0 | 1 | 1 | 1 | 1 | 0 | 1 | 7 |
| Mitra et al. (2000) | 1 | 1 | 0 | 1 | 1 | 1 | 1 | 0 | 1 | 7 |
| Sivri et al. (1996) | 1 | 1 | 0 | 0 | 1 | 1 | 1 | 0 | 1 | 6 |
| Donnelly et al. (1994) | 1 | 1 | 1 | 0 | 1 | 1 | 1 | 1 | 0 | 7 |
| Cooper et al. (1994) | 1 | 1 | 1 | 1 | 1 | 0 | 0 | 1 | 1 | 8 |
| Devogelaer et al. (1992) | 1 | 0 | 0 | 0 | 1 | 1 | 1 | 1 | 1 | 6 |
| Ralston et al. (1990) | 1 | 1 | 1 | 0 | 1 | 1 | 1 | 1 | 1 | 8 |

Quality assessment performed using the Joanna Briggs Institute critical appraisal tool for prevalence studies.

**Appendix E: Forest plot**
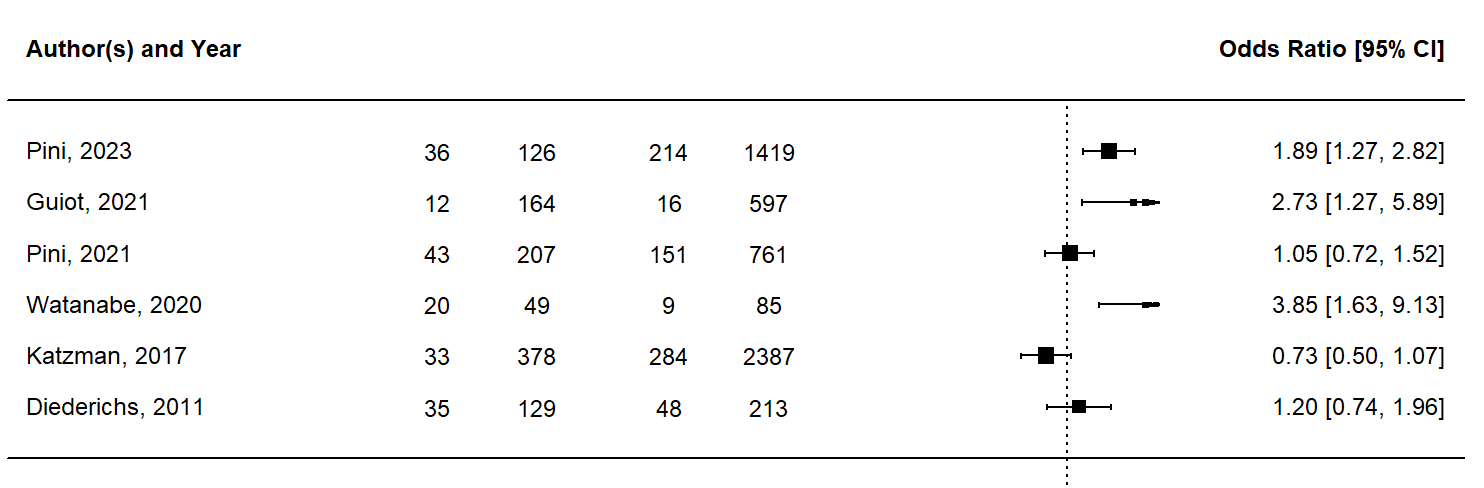


Supplementary Figure S1. Univariate odds ratio for vertebral fracture risk between DISH and non-DISH subjects

**Appendix F: Meta-regression and subgroup analyses for vertebral fractures in AS**


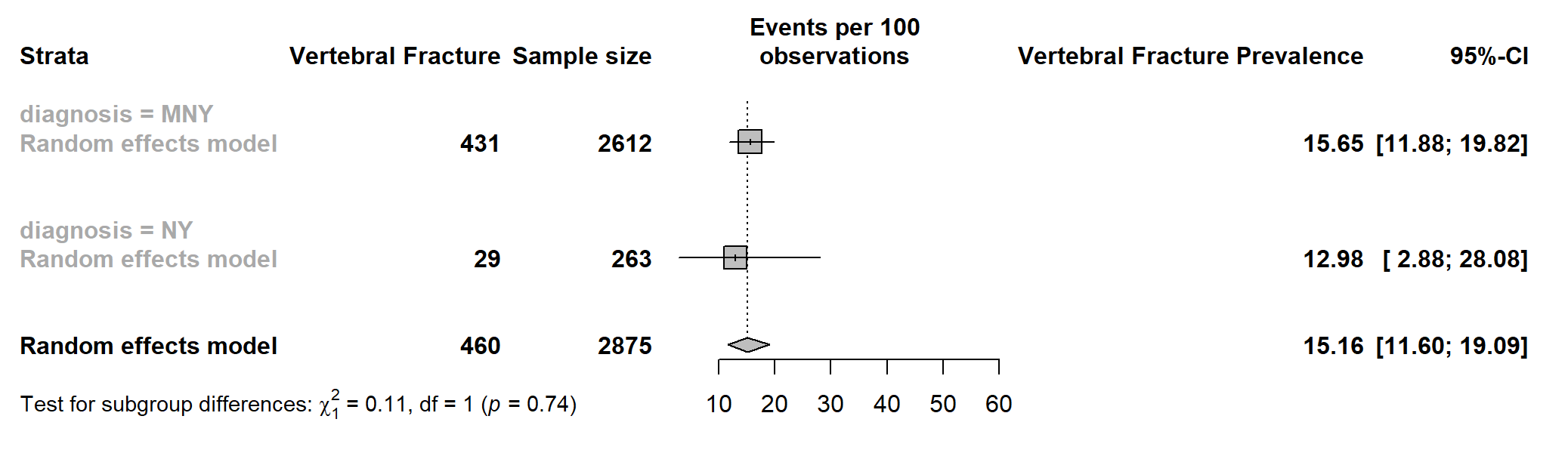


Meta-regression p-value = 0.57

Supplementary figure S2. Vertebral fracture rates in AS by diagnostic criteria.


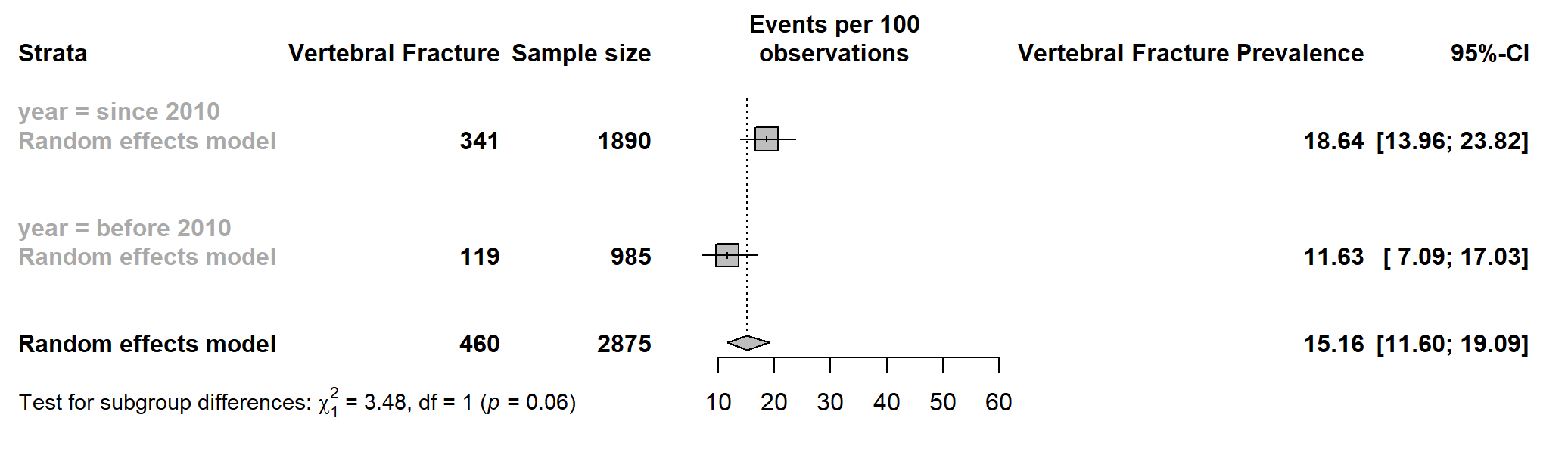


Meta-regression p-value = 0.06

Supplementary figure S3. Vertebral fracture rates in AS by publication year


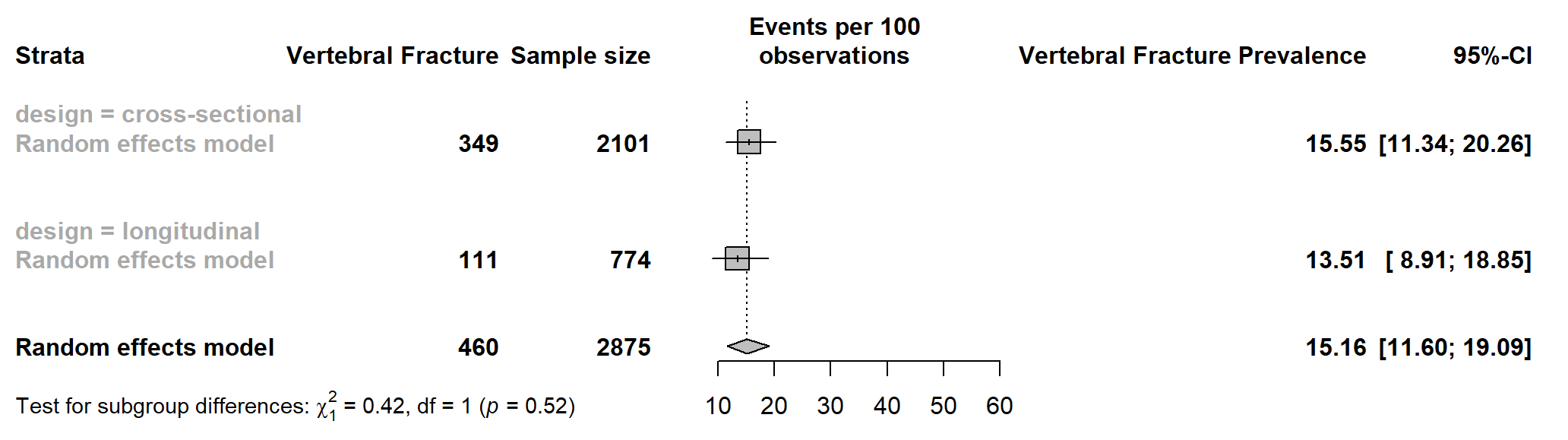


Meta-regression p-value = 0.65

Supplementary figure S4. Vertebral fracture rates in AS by study design


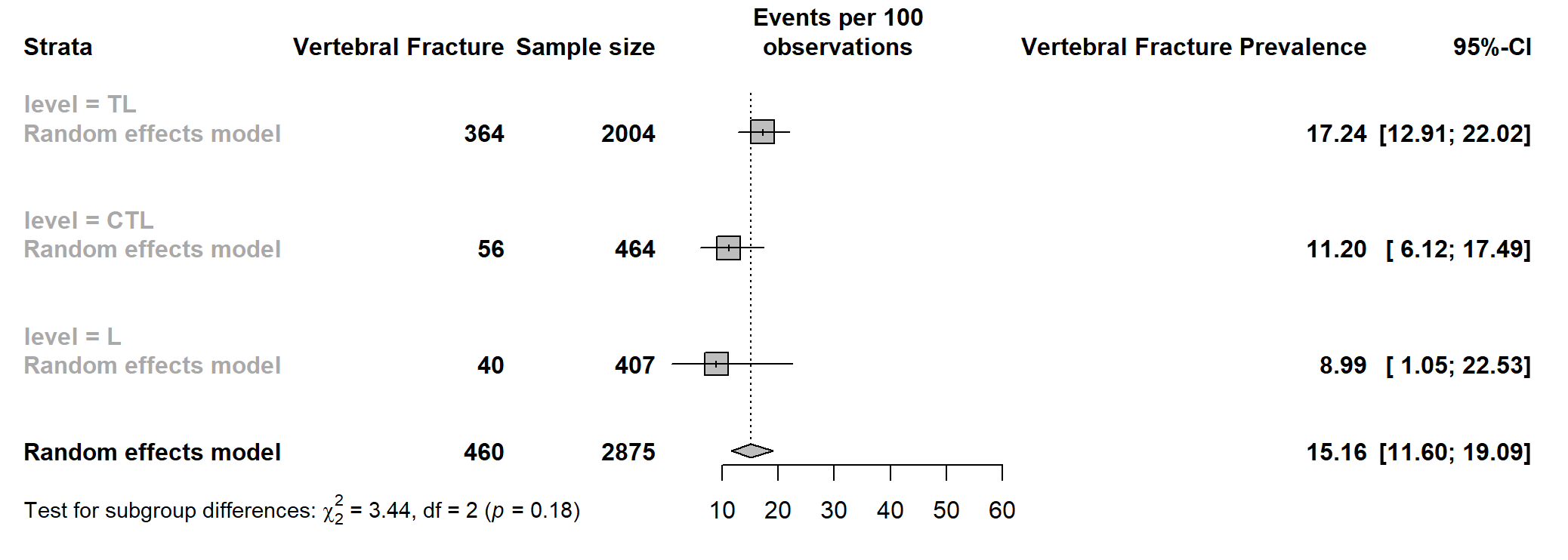


Meta-regression p-value = 0.19

Supplementary figure S5. Vertebral fracture rates in AS by spine level. TL: thoracolumbar spine; CTL: cervical, thoracic & lumbar spine; L: lumbar spine


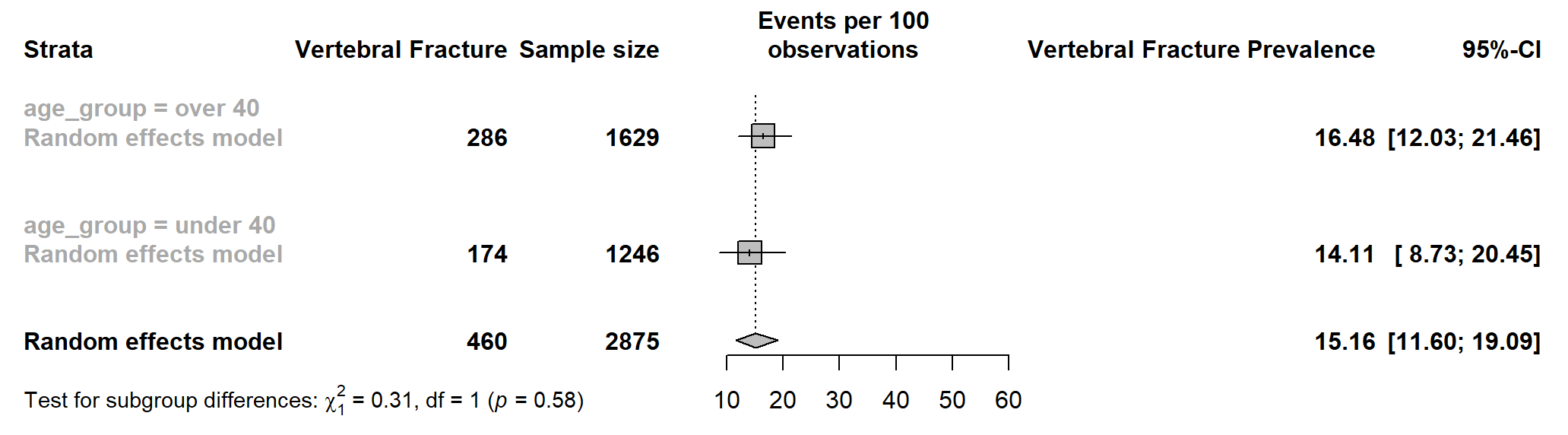


Meta-regression p-value = 0.56

Supplementary figure S6. Vertebral fracture rates in AS by age group


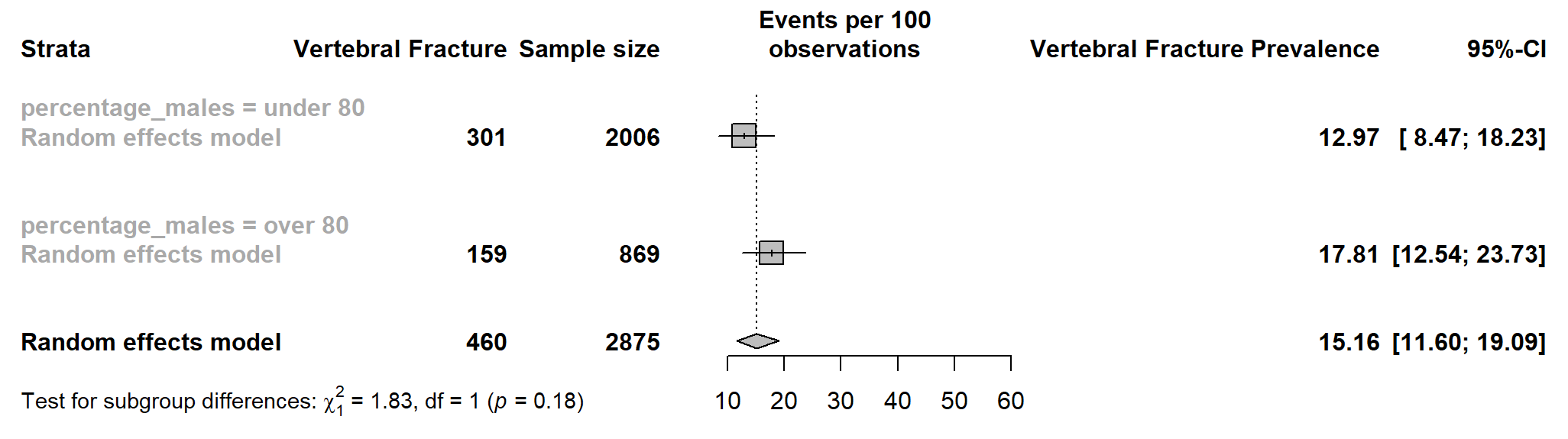


Meta-regression p-value = 0.18

Supplementary figure S7. Vertebral fracture rates in AS by percentage males


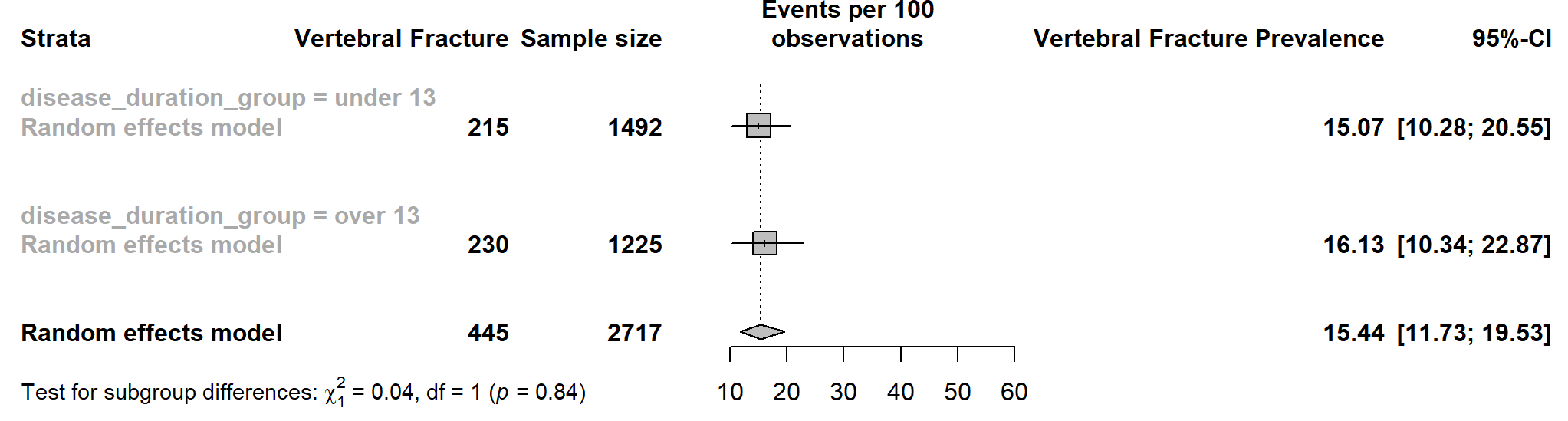


Meta-regression p-value = 0.84

Supplementary figure S8. Vertebral fracture rates in AS by disease duration >13 years (different values for vertebral fracture and sample size due to missing data)

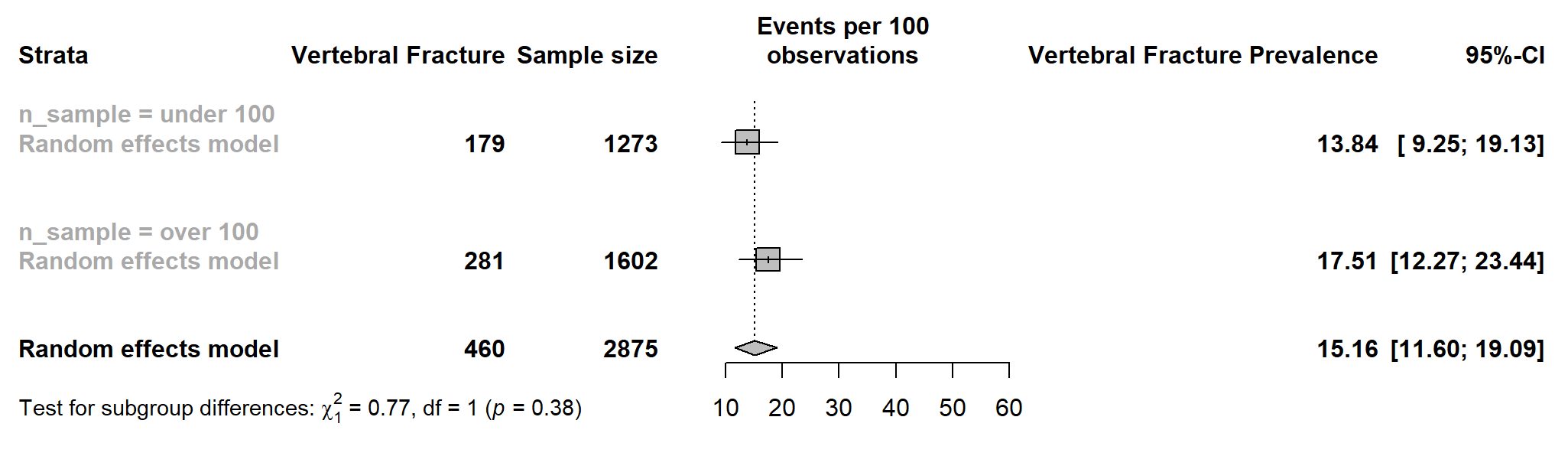


Meta-regression p-value = 0.39

Supplementary figure S9. Vertebral fracture rates in AS by sample size
